# Fibrous dysplasia of the jaw and diffuse large B-cell lymphoma coexistence, diagnosis and treatment experience and analysis: a case report

**DOI:** 10.3389/fsurg.2026.1928188

**Published:** 2026-09-25

**Authors:** Qing Hong, Chaowei Zhang, Weiyi Yao, Yuxuan Han, Yanan Zhu, Xinyu Tang, Na Chen

**Affiliations:** 1 Department of Hematology, Beijing Luhe Hospital, Capital Medical University, Beijing, China; 2 Department of Hematology, Shandong Provincial Hospital Affiliated to Shandong First Medical University, Jinan, Shandong, China; 3 Graduate School, Shandong First Medical University, Jinan, Shandong, China

**Keywords:** bone destruction, case report, chemotherapy, diffuse large B-cell lymphoma, fibrous dysplasia

## Abstract

**Background:**

Fibrous dysplasia (FD) is a rare benign fibro-osseous bone lesion, while diffuse large B-cell lymphoma (DLBCL) is the most common aggressive non-Hodgkin lymphoma. Their clinical and imaging manifestations overlap substantially, which complicates diagnosis, particularly when DLBCL manifests as extranodal bone lesions without peripheral lymphadenopathy. The coexistence of jaw FD and DLBCL is extremely rare, and relevant clinical experience is insufficient.

**Case description:**

This report presents a 46-year-old female with a 40-year history of jaw FD who presented with progressive right mandibular swelling and pain for 2 months. Laboratory tests showed elevated alkaline phosphatase. Imaging revealed right mandibular bone destruction, and pathological examination confirmed non-germinal center B-cell-like (non-GCB) DLBCL limited to the skeletal system, with double-expressor lymphoma features. The patient was staged as IIIA with an intermediate-low International Prognostic Index (IPI) and intermediate-high National Comprehensive Cancer Network International Prognostic Index(NCCN-IPI) risk. She received 8 cycles of polatuzumab vedotin, rituximab, cyclophosphamide, doxorubicin hydrochloride and prednisone (Pola-R-CHP) chemotherapy, achieving partial relief of symptoms and reduced mandibular mass, but residual lesions remained. Subsequently, second-line dexamethasone, high-dose cytarabine and cisplatin (DHAP) salvage therapy was initiated due to persistent disease activity.

**Conclusions:**

The coexistence of FD and DLBCL is easily misdiagnosed as FD progression. For FD patients with new-onset bone destruction and mass formation, timely pathological biopsy is essential to confirm malignancy. Accurate imaging, pathological evaluation, and risk stratification guide treatment selection. First-line chemoimmunotherapy may achieve partial remission in refractory cases, and second-line intensive salvage regimens are needed for persistent disease. This case provides a reference for the diagnosis and treatment of similar rare complex cases.

## 1 Introduction

Fibrous dysplasia (FD) is an uncommon fibro-osseous lesion, accounting for 5%-7% of all benign bone tumors, and is characterized by the replacement of normal bone architecture with fibrous tissue, resulting in structural abnormalities and functional impairment of the affected bone (1). FD is typically diagnosed during adolescence or early adulthood, with no marked sex predilection. The monostotic form accounts for 70%-80% of cases, whereas the polyostotic form is less frequently observed (2, 3). Craniofacial involvement occurs in 50%-100% of patients with polyostotic FD, most commonly affecting the frontal, sphenoid, ethmoid and maxillary bones (2, 3). Although the clinical course is often stable, a subset of patients may develop progressive bone destruction and local deformities (4, 5). FD may coincide with other neoplastic or dysplastic processes, particularly when the jaws are involved, and malignant transformation has been reported only in isolated cases (4, 5).

Diffuse large B-cell lymphoma (DLBCL) is the most common subtype of non-Hodgkin lymphoma (NHL), accounting for approximately one-third of all NHL cases worldwide (6). DLBCL is an aggressive malignancy that typically presents with lymphadenopathy, although extranodal bone involvement is well recognized (7, 8). This presentation shares features with primary lymphoma of bone (PLB), a rare extranodal lymphoma in which DLBCL is the most common histological subtype (9, 10). By integrating the clinical history of the patient, radiographic findings and pathological features, the present case report addresses a previously under-characterized clinical scenario, the coexistence of FD and DLBCL. The present case report underscores the importance of maintaining a high index of suspicion for malignancy in patients with FD presenting with osteolytic progression, and contributes valuable insights to the clinical management of such complex cases.

## 2 Case presentation

A 46-year-old woman, employed as a farmer, with a 40-year history of FD presented with worsening right mandibular swelling over 2 months, accompanied by local pain. The patient showed little clinical improvement after taking oral ibuprofen sustained-release capsules (0.3 g per capsule, twice daily) for 5 days. The patient was admitted to Shandong Provincial Hospital Affiliated to Shandong First Medical University (Jinan, China) in January 2025.

The patient exhibited facial asymmetry. The range and pattern of mouth opening were acceptable, and there was no popping or tenderness in either temporomandibular joint area. The right mandibular gland was notably enlarged, and a soft, poorly defined 6.0 × 6.0 cm mass was palpable with normal skin color and slightly elevated skin temperature. The intraoral examination revealed missing teeth (specifically, the upper left second molar and lower right first molar), a residual root (the lower left central incisor), and severe tooth wear affecting multiple teeth (including the upper left canine and first premolar, the lower right central and lateral incisors, the lower right second premolar, and the lower right second molar). There was a slight mandibular deviation, with a leftward shift of approximately 1.0 cm at the midline. The alveolar bone of the upper and lower right jaw exhibited buccal expansion, and the palatal bone showed hyperplasia. No lymphadenopathy or other systemic symptoms were noted. The patient did not report any fever, night sweats or weight loss.

Routine blood tests and biochemical tests were within normal limits, with the exception of elevated alkaline phosphatase levels (338 U/L; normal range: 35–100 U/L), which indicated bone metabolism abnormalities. Computed tomography(CT) of the skull base to neck root showed bone destruction in the right mandible with osteopenia in some regions and periosteal reaction (Figure 1A). Combined with the patient's FD history, progressive mandibular swelling and CT osteolytic lesions, we systematically completed differential diagnosis before biopsy to exclude five mimicking disorders. Mandibular osteomyelitis, FD-associated sarcoma, metastatic bone tumor, primary bone sarcoma and reactive fibrous hyperplasia were all ruled out via clinical symptoms, imaging and preliminary laboratory evidence. After exclusion, hematological malignancy was highly suspected, and surgical biopsy was performed for pathological confirmation. In January 2025, the patient underwent incision and drainage for a right-sided mandibular abscess at the oral and maxillofacial surgery department in our hospital. Intraoperative frozen section pathology indicated a poorly differentiated malignant tumor (Figure 1B), and the patient was subsequently referred to Hematology Department in our hospital for further treatment. Postoperative pathological examination revealed an aggressive B-cell lymphoma (Figure 1C). Immunohistochemical staining (Figure 2A–C) demonstrated that the tumor cells were positive for CD20, paired box 5 (PAX5) and multiple myeloma oncogene 1 (MUM1), confirming a B-cell lineage. The immunophenotype (CD10^−^, BCL-6^−^ and MUM1^+^) (Figure 2C–E) was consistent with the non-germinal center B-cell-like (non-GCB) subtype according to the Hans algorithm (11). Furthermore, the tumor cells exhibited co-expression of BCL-2, MYC (+70%) and a high Ki-67 proliferation index of 80% (Figure 2F), indicative of double-expressor lymphoma features, which are generally associated with an unfavorable prognosis (11). Bone marrow aspiration and smear cytology revealed no infiltration by lymphoma cells.

**Figure 1:**
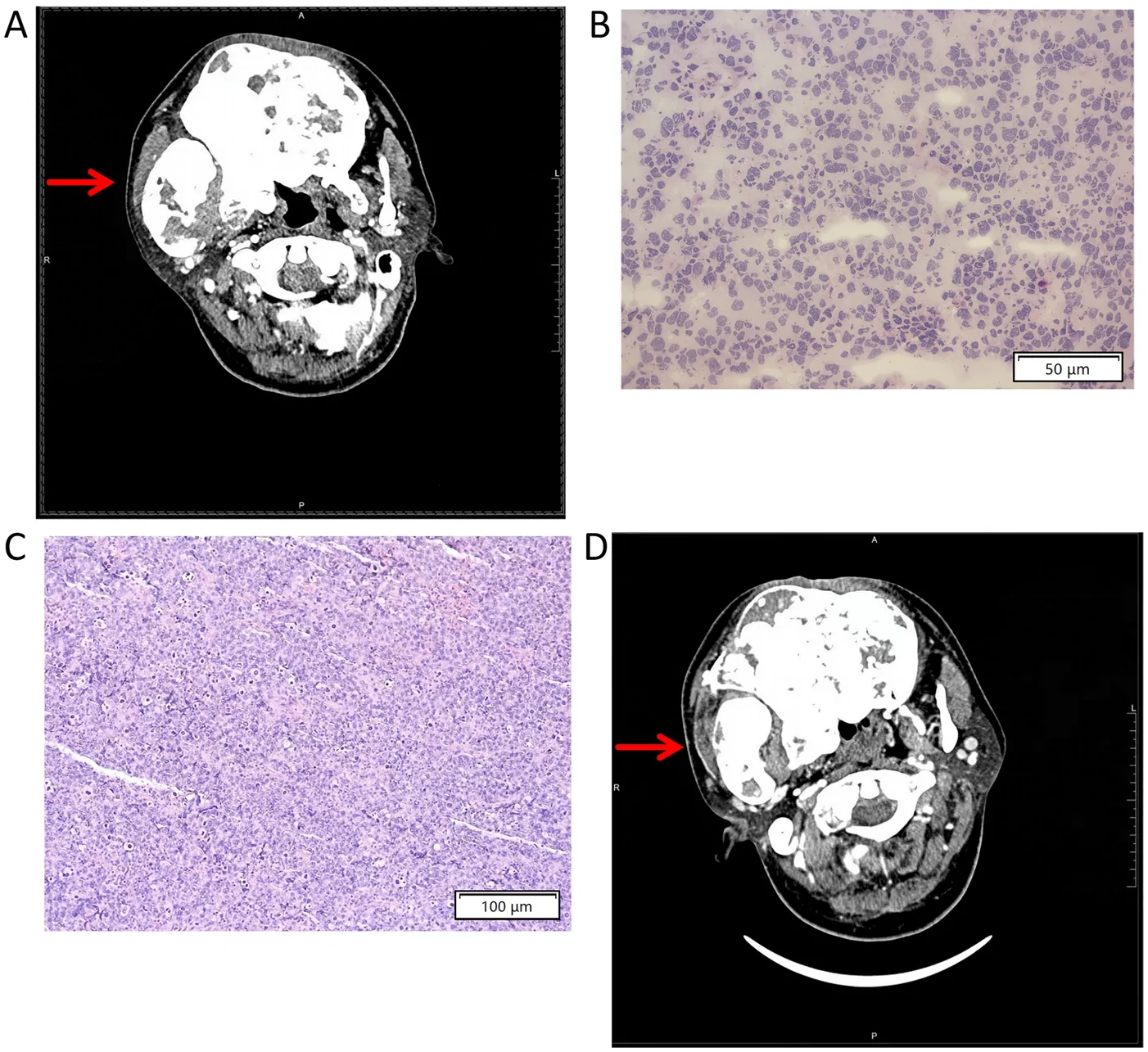
**(A)** CT performed in January 2025 showing bone destruction of the right mandible with osteopenia in some regions and periosteal reaction; the red arrow indicates the right mandibular mass lesion. **(B)** Intraoperative frozen section pathology indicating a poorly differentiated malignant tumor (magnification, ×20). **(C)** Postoperative pathological examination revealing an aggressive B-cell lymphoma (magnification, ×10). **(D)** Follow-up CT performed in September 2025 showing persistent involvement of the right mandibular lesion; the red arrow indicates the right mandibular mass lesion. This image was published with the patient's written informed consent.

**Figure 2:**
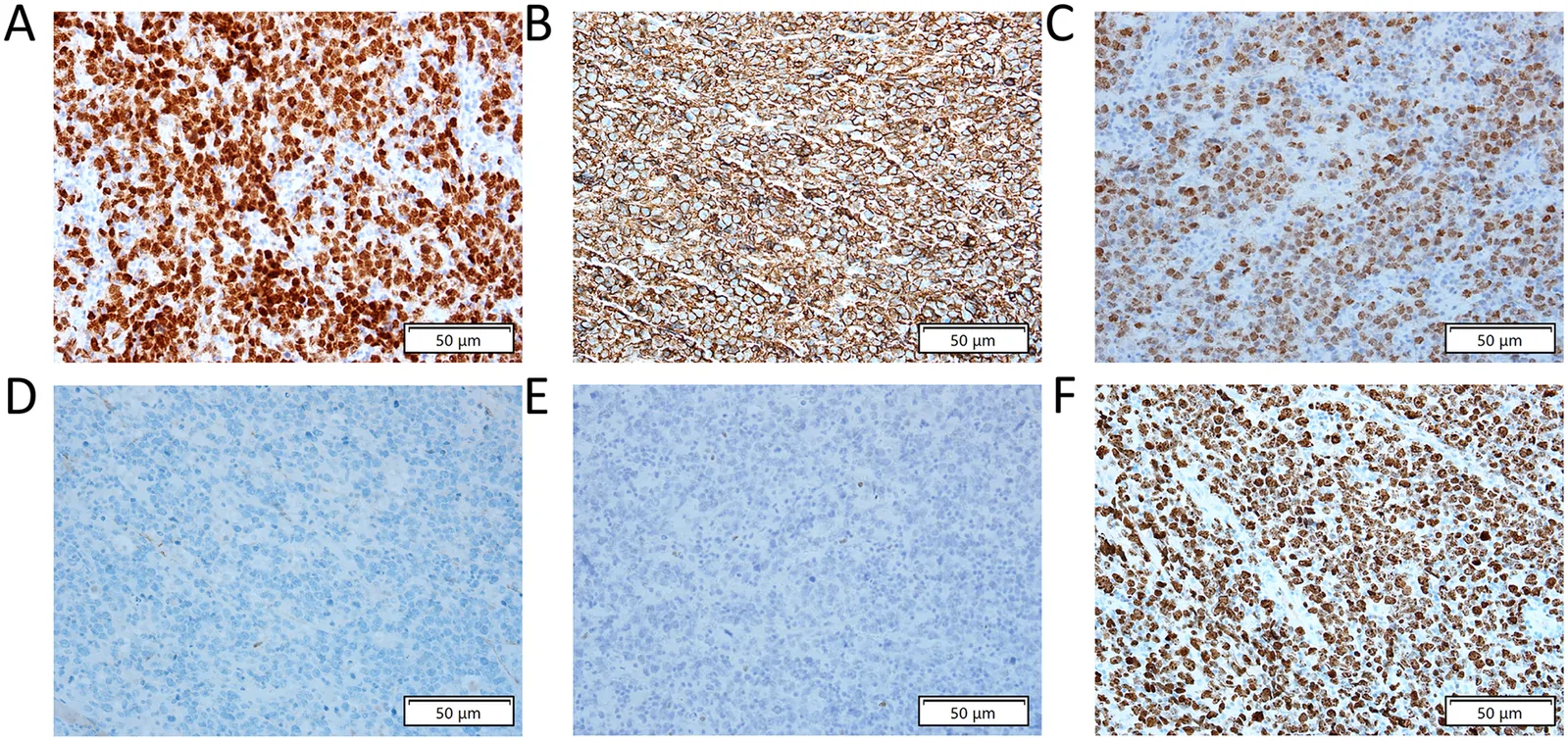
**(A)** PAX5 immunohistochemistry (IHC) (magnification, ×20). The tumor cells were positive for PAX5, exhibiting diffuse nuclear staining. **(B)** CD20 IHC (magnification, ×20). The tumor cells were positive for CD20, exhibiting diffuse and strong membranous expression. **(C)** MUM1 IHC (magnification, ×20). The tumor cells were positive for MUM1, exhibiting diffuse nuclear staining. **(D)** CD10 IHC (magnification, ×20). The tumor cells were negative for CD10 **(E)** BCL-6 IHC (magnification, ×20). The tumor cells were negative for BCL-6. **(F)** Ki-67 IHC (magnification, ×20). Approximately 80% of the tumor cells were positive for Ki-67, exhibiting diffuse nuclear staining, indicating a high proliferative index. IHC, immunohistochemistry; MUM1, multiple myeloma oncogene 1; PAX5, paired box 5. This image was published with the patient's written informed consent.

Bone marrow examination, including aspiration cytology and biopsy, showed no evidence of lymphoma involvement. Since the patient refused ¹⁸F-fluorodeoxyglucose positron emission tomography/computed tomography (¹⁸F-FDG PET/CT), routine chest and abdominal CT scans were performed, and the report showed bone destruction in the jaw, thoracic vertebrae, pubis, femur and bilateral ischia without lymphadenopathy. In the present case, the patient had a long-standing history of FD; however, postoperative histopathological examination of the mandibular mass revealed coexistent DLBCL. Comprehensive staging investigations demonstrated no evidence of lymph node involvement, indicating that the disease was confined to the skeletal system. The final diagnosis was DLBCL (non-GCB type; stage III; group A) (11). According to the International Prognostic Index (IPI) (11) for DLBCL, the score was 2, categorizing the patient as medium-low risk. According to the National Comprehensive Cancer Network (NCCN)-IPI (11) score, the risk was medium-high.

Given the refractory nature of the disease, the patient received standard polatuzumab vedotin, rituximab, cyclophosphamide, doxorubicin hydrochloride and prednisone (Pola-R-CHP) chemotherapy for three cycles (580 mg polatuzumab vedotin and 90 mg rituximab administered on Day 1; 1.1 g cyclophosphamide and 40 mg doxorubicin on Day 2; 100 mg prednisone daily from Days 1 to 5) for three cycles. After three cycles of chemotherapy,PET/CT (Figure 3A,B) revealed multiple bone destructive lesions, including the skull, maxillofacial bones, left ribs, right ulna, right finger bones, right femur, spine and pelvis, with intense fluorodeoxyglucose (FDG) avidity, suggesting post-treatment lymphoma activity. To evaluate the etiology of multifocal bone destruction throughout the body and its correlation with lymphoma, percutaneous needle biopsy of the right femoral lesion was performed in April 2025. Postoperative pathological examination favored fibrous dysplasia with no atypical lymphocytes identified. Repeat bone marrow morphology showed no infiltration of lymphoma cells.

**Figure 3:**
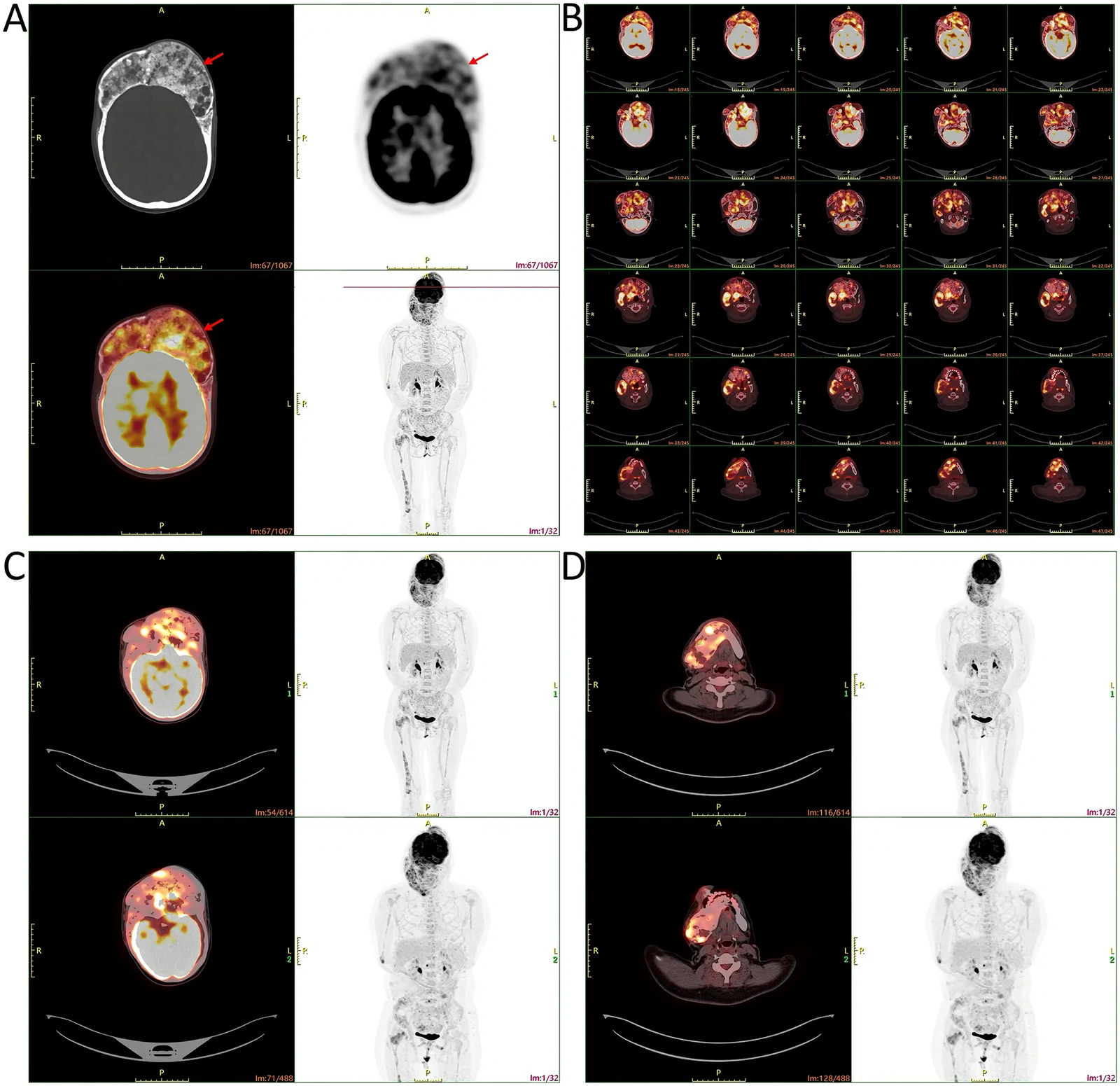
**(A)** Baseline axial CT, PET and fused PET/CT images (April 2025). The native red arrow points to the primary hypermetabolic cranial fibro-osseous lesion with intense radiotracer uptake. **(B)** Baseline multiplanar fused PET/CT cross-sections of the head and neck, showing extensive abnormal FDG accumulation within the cranial lesion. **(C,D)** Paired axial fused PET/CT images of the cranial and mandibular region acquired after 3 cycles (upper panels, April 2025) and 6 cycles (lower panels, June 2025) of Pola-R-CHP chemoimmunotherapy. Sequential scans reveal obvious shrinkage of lesion volume and remarkable decline in intralesional ¹⁸F-FDG avidity, demonstrating favorable therapeutic response localized to the cranial and mandibular lesions.

After the patient had completed an additional three cycles of the same Pola-R-CHP regimen following the interim evaluation after the initial three cycles, a follow-up PET/CT was performed in June 2025 (Figure 3C,D). The scan showed widespread bone destruction with mixed lytic and osteoblastic changes, with reduced FDG uptake in the jaw, right hand fingers and lumbar vertebra 5 (L5), but increased uptake in the skull, right femur, right ulna and right iliac bone. The mandibular mass shrank, and pain relief was noted. By September 2025, the patient had finished eight cycles of Pola-R-CHP chemotherapy with favorable tolerance. Although the clinical symptoms including maxillofacial pain and swelling improved and the maxillofacial lesions decreased, the current regimen failed to eradicate all lesions, and follow-up CT revealed persistent right mandibular disease (Figure 1D).

Therefore, further exploration of second-line treatments is needed. Due to the persistent activity, salvage therapy with dexamethasone, high-dose cytarabine and cisplatin (DHAP) regimen was initiated, consisting of cisplatin at 100 mg/m² as a continuous 24-h infusion on Day 1, cytarabine (Ara-C) at 2 g/m² infused over 3 h every 12 h for two doses on Day 2, and dexamethasone at 40 mg on Days 1-4. A standardized long-term surveillance strategy was established for this patient: during salvage treatment, whole-body enhanced CT, physical examination and serological tests should be performed every two cycles. After second-line chemotherapy, PET/CT surveillance may be scheduled every 3 months in the first two years alongside routine physical and laboratory assessments. From year 3 to year 5, PET/CT could be arranged semi-annually. After 5 years, annual contrast-enhanced whole-body CT combined with serological screening can be used, and PET/CT might be carried out if suspicious lesions emerge. Serum alkaline phosphatase and lactate dehydrogenase may be measured at each follow-up to monitor bone lesion activity and lymphoma recurrence.

## 3 Discussion

DLBCL usually manifests as lymphadenopathy (6), but this patient only had bone-limited lesions, bringing diagnostic difficulties. FD and osseous DLBCL show highly overlapping imaging features on CT, MRI and ¹⁸F-FDG PET/CT. On non-contrast CT, both display expansile fibro-osseous lesions with ground-glass density, thin cortices and focal osteolysis; MRI fails to differentiate them due to identical heterogeneous T1/T2 signals and irregular enhancement (12). Notably, chronic inflammation in FD causes intense FDG uptake on PET/CT, often leading to false-positive staging misdiagnosed as widespread lymphoma (13). Previous cases misclassified benign FD hypermetabolism on PET/CT as progressive bone lymphoma, resulting in excessive chemotherapy (14). Our mandibular lesion possessed all these confusing imaging traits: DLBCL glycolysis overlapped FD inflammatory metabolism on PET/CT, making reliable radiological discrimination difficult. Thus, histopathological biopsy is indispensable to confirm coexisting FD and lymphoma within one bone lesion, instead of depending merely on imaging scans.

For FD patients presenting with rapid osteolytic lesions and soft tissue masses, five key differential diagnoses need to be excluded based on clinical, imaging and pathological evidence. (1)Mandibular osteomyelitis: Systemic fever, elevated inflammatory markers, bone sequestra and sinus tracts were absent in this patient, excluding osteomyelitis. (2)FD-associated secondary sarcoma: malignant transformation of FD mostly produces osteosarcoma or angiosarcoma with typical cloud-like osteoid matrix and Codman triangles on CT. No sarcomatous components were identified in our pathological samples. (3)Metastatic bone tumor: Whole-body CT detected no primary epithelial malignancies, and femoral biopsy showed no epithelial tumor cells, ruling out bone metastasis. (4)Primary bone sarcoma: Tumor cells were diffusely positive for B-cell markers CD20/PAX5 and negative for mesenchymal markers, excluding chondrosarcoma and fibrosarcoma. (5)Chronic reactive fibro-osseous hyperplasia: Diffuse atypical lymphoid infiltration and high Ki-67 index indicated malignant lymphoproliferative disease rather than benign reactive lesions. Most reported FD-complicated malignancies are sarcomas (Table 1), while FD combined with hematological tumors is rarely documented. This non-GCB DLBCL case provides rare clinical evidence.

**Table 1:** Summary of published malignant tumors coexisting with fibrous dysplasia.

Tumor classification	Specific pathological type	Clinical & imaging characteristics	Supporting references
Sarcoma (malignant transformation arising inside FD lesions)	Osteosarcoma	Most common FD malignant transformation subtype; mainly craniofacial/long bone involvement; CT shows cloud-like osteoid matrix and Codman triangle periosteal reaction	(3, 15)
	Fibrosarcoma	Secondary to polyostotic FD; rapid bone swelling and pain, no characteristic osteoid matrix on imaging	(2, 3)
	Chondrosarcoma	Rare FD-associated sarcoma, mostly occurs in monostotic FD of long bones, with irregular chondroid matrix on CT	(2, 3)
	Angiosarcoma	Rare FD malignant transformation subtype; high risk of pathological fracture and distant metastasis, poor overall survival	(1)
Incidental separate-site hematological malignancy (FD and lymphoma locate in different bones, no intra-lesional coexistence)	Classical Hodgkin lymphoma	FD and nodal Hodgkin lymphoma occur independently in distinct bones; FDG hypermetabolism of FD leads to false-positive staging misjudgment	(14, 16)
	Intestinal non-Hodgkin lymphoma	Primary intestinal lymphoma, incidental spinal FD detected during staging PET; FD hypermetabolism mimics bone lymphoma infiltration	(17)

Summary of pathological subtypes, clinical and imaging manifestations, and supporting literature of all malignant tumors reported to coexist with fibrous dysplasia. This table is divided into two major groups: sarcoma derived from malignant transformation of fibrous dysplasia, and incidental hematological malignancies.

The lesion phenotype of this case differs fundamentally from malignant transformation of fibrous dysplasia at the mechanistic level. Malignant transformation of fibrous dysplasia refers to sarcomatous degeneration of the native FD stroma, whereas no sarcomatous components or premalignant dysplastic lesions were detected in FD tissues harvested from our patient. Histological evaluation of mandibular biopsy specimens suggests that DLBCL represents an independent hematological malignancy. It develops within the chronic inflammatory microenvironment of pre-existing FD lesions and secondarily invades fibro-osseous tissues, rather than arising from malignant degeneration of FD itself.

Chronic bone disruption and local immune dysregulation in FD not only limit skeletal function but may also alter the local immune microenvironment, potentially providing a “biological platform” that might contribute to malignancies such as DLBCL, based on published literature. Activating GNAS mutations reshape FD stroma into a tumor-prone inflammatory niche, presumably distinct from FD sarcomatous transformation (15, 18). No premalignant or sarcomatous components were found in our patient's bone samples, suggesting that DLBCL secondarily infiltrated FD lesions rather than deriving from FD stromal malignant transformation. Mutant bone progenitors overproduce RANKL to trigger bone loss and persistent inflammation. Sustained IL-6, TNF-α and IL-1β may activate B-cell NF-*κ*B and JAK/STAT3 pathways (19), possibly driving malignant proliferation and immune escape, while FD stroma recruits suppressive immune cells to weaken anti-tumor responses. Unlike prior cases with separate lesions, DLBCL arose within FD tissue here. We speculate the remodeled inflammatory niche fuels B-cell expansion (20). DLBCL in turn upregulates RANKL/VEGF to aggravate osteolysis, forming a vicious cycle that may partly explain the rapid bone destruction observed clinically. Combined GNAS mutation, chronic inflammation, disrupted bone niche and impaired immunity may account for their rare synchronous coexistence in one bone lesion (21, 22). Still, these causal links cannot be validated by a single case; further research is needed to confirm the hypotheses.

Published clinical data indicate that non-GCB subtype DLBCL with double-expressor phenotype carries an unfavorable prognosis (6, 11). Patients with isolated primary bone DLBCL commonly achieve complete remission after standard R-CHP-like regimens; however, cases with disseminated multifocal bone involvement frequently (9)only attain partial response and necessitate subsequent salvage therapy, which matches the treatment response observed in our case. Concurrent long-standing fibrous dysplasia may increase the risk of skeletal complications, which could potentially exert an unfavorable influence on long-term survival, though reliable survival comparison data specific to FD combined DLBCL are still scarce (15, 18).

Per NCCN guidelines (11) persistent active disease prompted an immediate switch to second-line salvage therapy. CAR-T (lisocabtagene maraleucel) and tafasitamab plus lenalidomide are preferred second-line options, yet selection requires full consideration of the tumor's non-GCB subtype and widespread bone infiltration. These targeted and cellular immunotherapies have no dedicated clinical data for patients with long-term FD, so their efficacy and safety in this special group remain unconfirmed and require prospective research for validation. Given the above factors, we adopted the intensive DHAP salvage regimen, a standard choice for aggressive lymphoma refractory to frontline chemoimmunotherapy (11). Clinically, we aim for deep remission; bone-modifying agents may be added later to lower risks of skeletal complications from diffuse bone lesions.

Future research should further explore the relationship between immune regulation, gene mutations and the microenvironment to elucidate their potential roles in the coexistence of FD and DLBCL. Such insights will aid clinicians in differentiating between benign and malignant lesions. Additionally, as targeted therapies and immunotherapies continue to advance, their application in complex cases, such as the present case, could offer more individualized and precise therapeutic options.

## 4 Conclusions

The present case report highlights the complexity of the coexistence of DLBCL and FD, particularly when imaging and clinical symptoms overlap. Pathological examination is key in these cases. For patients with FD with bone destruction, the possibility of malignant lymphoma should be considered, and timely imaging and biopsy are essential for accurate diagnosis and treatment. As research on the microenvironment, immune regulation and gene mutations progresses, personalized treatment options may provide more support in the future. Early diagnosis and standardized individualized treatment may help optimize clinical outcomes for such complex patients. Novel targeted immunotherapies could represent promising exploratory therapeutic directions for cases with concurrent FD and disseminated bone-involved DLBCL, yet further clinical research is required to confirm their efficacy.

## Data Availability

The original contributions presented in the study are included in the article/Supplementary Material, further inquiries can be directed to the corresponding author.
